# Refining complex re‐irradiation dosimetry through feasibility benchmarking and analysis for informed treatment planning

**DOI:** 10.1002/acm2.13102

**Published:** 2020-12-03

**Authors:** Seth R. Duffy, Yiran Zheng, Jessica Muenkel, Rodney J. Ellis, Tanvir N. Baig, Brian Krancevic, Christian B. Langmack, Kevin D. Kelley, Serah Choi

**Affiliations:** ^1^ Radiation Oncology University Hospital Cleveland Medical Center Cleveland OH USA; ^2^ Radiation Oncology Penn State Health Hershey PA USA

**Keywords:** dosimetry, innovation, pinnacle, planIQ, radiotherapy, re‐irradiation

## Abstract

**Purpose/Objectives:**

The purpose of this study is to dually evaluate the effectiveness of PlanIQ in predicting the viability and outcome of dosimetric planning in cases of complex re‐irradiation as well as generating an equivalent plan through Pinnacle integration. The study also postulates that a possible strength of PlanIQ lies in mitigating pre‐optimization uncertainties tied directly to dose overlap regions where re‐irradiation is necessary.

**Methods:**

A retrospective patient selection (*n* = 20) included a diverse range of re‐irradiation cases to be planned using Pinnacle auto‐planning with PlanIQ integration. A consistent planning template was developed and applied across all cases. Direct plan comparisons of manual plans against feasibility‐produced plans were performed by physician(s) with dosimetry recording relevant proximal OAR and planning timeline data.

**Results and Discussion:**

All re‐irradiation cases were successfully predicted to be achievable per PlanIQ analyses with three cases (3/20) necessitating 95% target coverage conditions, previously exhibited in the manually planned counterparts, and determined acceptable under institutional standards. At the same time, PlanIQ consistently produced plans of equal or greater quality to the previously manually planned re‐irradiation across all (20/20) trials (*P* = 0.05).

Proximal OAR exhibited similar to slightly improved maximum point doses from feasibility‐based planning with the largest advantages gained found within the subset of cranial and spine overlap cases, where improvements upward of 10.9% were observed. Mean doses to proximal tissues were found to be a statistically significant (*P* < 0.05) 5.0% improvement across the entire study. Documented planning times were markedly less than or equal to the time contributed to manual planning across all cases.

**Conclusion:**

Initial findings indicate that PlanIQ effectively provides the user clear feasibility feedback capable of facilitating decision‐making on whether re‐irradiation dose objectives and prescription dose coverage are possible at the onset of treatment planning thus eliminating possible trial and error associated with some manual planning. Introducing model‐based prediction tools into planning of complex re‐irradiation cases yielded positive outcomes on the final treatment plans.

## 1 Introduction

Epidemiologists have described the United States population as one that is aging and where advances in medicine and greater investments in the study of public health have not only improved quality of life but have also extended it.^1^ Concomitantly, anecdotal evidence suggests that clinicians in radiation oncology experience a phenomenon of patients returning for multiple curative intent courses of radiotherapy. Patients who do require complex dosimetric planning, such as those receiving a second course of treatments overlapping the same anatomical site, require techniques and approaches that are equally flexible in considering and achieving each individual patient’s needs. Specifically within the dosimetric planning of these complex re‐irradiation cases, PlanIQ (Sun Nuclear Corp, Melbourne, FL) feasibility benchmarking and analyses make it possible to visualize and appreciate possible dosimetric outcomes prior to in‐depth optimization, thus providing valuable insight for physician decision‐making. Pinnacle auto‐planning (AP) (Philips Medical Systems, Fitchburg, WI) optimization algorithms, mirroring techniques used by experienced medical dosimetrists, make it possible to translate the model‐based benchmarking seen in PlanIQ feedback into tangible, treatable, radiotherapy plans that may not otherwise be achievable through historical means of treatment optimization.^2^

The role of radiation within the cancer care continuum has become increasingly well understood in the 20^th^ and 21^st^ centuries, with studies suggesting a clinical indication for irradiation in over 50% of all diagnosed cases.^3^, ^4^ Furthermore, the advent and commercial use of treatment planning solutions, such as multi‐criteria optimization (MCO), knowledge‐based planning, AP, and PlanIQ, have enhanced practitioner’s decision‐making and reduced inter‐ and intra‐planner variation.^5^ Clinical researchers have also demonstrated powerful applications for PlanIQ and automated planning in cases of head and neck, hippocampal sparing, and stereotactic ablative body radiotherapy (SBRT),^6^, ^7^, ^8^. However, a gap in literature illustrating the true breadth of cases in which these treatment planning solutions are applicable has been identified. Consequently, this paper postulates the idea that a possible strength of PlanIQ rests in its ability to mitigate pre‐optimization uncertainties tied directly to the dose overlap regions in cases of re‐irradiation, presenting the planner with meaningful choices on how to improve plan optimization through the feasibility of the various constraints dosimetrists provide the software.

Purposely designed to replicate the actions taken by experienced planners, Pinnacle’s AP has been shown to generate clinically acceptable treatment options by leveraging an iterative approach to optimization.^6^, ^9^ Additionally, AP allows users to develop templates, referred to as *treatment techniques*, for generalizable planning scenarios upon which planners may manually optimize and/or adjust at a later time. Manual adjustment, in this sense, is often exhibited in trial and error efforts to achieve a satisfactory outcome. An important limitation to note, as illustrated by Kumar^10^ and Esposito,^11^ is that AP quality is highly dependent on the initial user request(s) and/or planner experience. In isolation, AP does not eliminate the uncertainty of whether a clinical objective is achievable or not. As a focal point within this study, the uncertainties of re‐irradiation further exacerbate the limitations of automated planning functionality.

Representing the second core component within this research, PlanIQ offers a robust means of assessing patient anatomy, idealistic dose distributions, and clinical objectives prior to optimizing within a TPS.^12^ Considered a highly valuable and validated tool for dosimetric planning, Plan IQ utilizes a priori estimation of each clinical goal, returning a feasibility score that planners can interpret, mitigating historical uncertainties, and optimization backtracking.^13^, ^14^ More specifically, PlanIQ returns users’ optimization objectives as values along a *feasibility dose–volume histogram* (FDVH) that can be examined and/or manipulated to achieve ideal sparing of proximal tissues through Pinnacle optimization.^15^ Recently, this has become a seamless process, as Pinnacle autoplan now offers direct export and import options for PlanIQ. Therefore, it is at the intersection of these two treatment planning solutions that we begin to develop a newfound sense of informed treatment planning for cases of re‐irradiation.

## 2 Purpose

The purpose of this study is to dually evaluate the effectiveness of PlanIQ in predicting the outcome of dosimetric planning in cases of complex re‐irradiation as well as generating a treatment plan of equivalent quality through Pinnacle AP integration. Through the model‐based system PlanIQ incorporates in its dose calculation methods, planners are presented with meaningful choices via PlanIQ *Dose Analysis and Benchmarking* from which this study attempts to integrate and refine the processes involved in the treatment planning of complex re‐irradiation cases.

Historically, planning of re‐irradiation cases has proven to be a time‐consuming process for both physicians and planners alike.^16^ Often times, trial and error plays a large role in determining dose objectives that are achievable within a planning system based solely on each individual planner’s results for a given iteration of the optimization process,^17^ whereas PlanIQ now looks to mitigate pre‐optimization uncertainties in defining objectives and providing much needed clarity for dosimetrists through feasibility histograms. Specifically, in cases of re‐irradiation where the dose‐overlap regions directly affect the decision‐making process of both physicians and treatment planners, dose feasibility benchmarking may provide the insight necessary to eliminate the trial and error of blind optimization all together. As a novel approach to examining and generating a re‐irradiation treatment plan, the integration of PlanIQ and AP allows for informed treatment planning that was previously inaccessible and/or based on trial and error. Not unlike earlier suggestions, the scope of this investigation is not to determine the finite best planning solution but rather to examine PlanIQ and its ability to enhance the dosimetrists planning knowledge through a system wherein guidance on achieving dose objectives is clearly relayed to the user as a metric of feasibility.^18^

This study also aims to provide direct DVH comparisons and statistical analyses between the initial manually planned outcomes and the AP/PlanIQ outcomes. These comparisons are valuable because all of the plans evaluated within PlanIQ have been previously achieved through manual planning alone whereas this study now looks to refine and advance the process of planning re‐irradiation through the use of AP and feasibility software. As previously noted by Fried et al.,^13^ complex treatment planning has an inherent degree of variability that rests largely with the user and their interpretation of how delineated target volumes and dose objectives interact. In the novel examination of re‐irradiation cases, the integration of AP and PlanIQ mitigates the user‐related variability with respect to previously delivered dose and the perceived influence of that dose with newly outlined treatment objectives. This perception leads to blind optimization in the absence of a feasibility planning solutions such as PlanIQ, which this study believes may exacerbate an already challenging planning scenario.

## 3 Methods

### 3.A Patient selection & setup

Patient selection (*n* = 20) included a diverse range of re‐irradiation cases from University Hospitals Seidman Cancer Center in Cleveland, Ohio. Re‐irradiation cases, ranging from the brain to the lower extremities, are expected to provide an examination of the scope of applications in which feasibility benchmarking is applicable, rather than a snapshot of one specific anatomical site. All patients were simulated head first supine using a Siemens Somatom Sensation CT scanner (Siemens Medical Solutions, Malvem, PA) using 1–3 mm slice thickness. Immobilization techniques varied based on the desired site of treatment and patient reproducibility.

### 3.B Target delineation

Pinnacle TPS (version 14.0 ‐ 16.2) and MIM (version 6.6) (MIM Software, Cleveland, OH) were used by the attending physician(s) to delineate both normal organs at risk (OAR) and approved target volumes. Variable target margins were used at the physician’s discretion due to anatomical restrictions and proximity to sites of prior irradiation.

Dosimetry contouring was minimal and consisted primarily of segmenting OAR based on previously delivered doses which would then be exported to PlanIQ. These optimization target volumes, generated to account for direct overlap with previously irradiated critical structures, were used in two (2/20) cases to achieve a plan of equal quality with the manual plan. Given the prior treatment planning that had occurred, normal OAR were not modified and were evaluated as they were at the time of initial planning review. Artifacts produced by metallic implants, fillings, or fiducials were manually assigned a density matching that of the nearby tissues, bone, or adipose.

### 3.C Treatment planning

Using Pinnacle (version 16.2), MIM (version 6.6), and PlanIQ (version 2.2), the dosimetrist imported the composite dose from any prior treatments to the new treatment planning CT (TPCT). Regions of overlap were determined, and based on the physician’s outlined treatment objectives, an initial AP was generated for export to PlanIQ. From the original plan, patient‐specific prescriptions and dose constraints dictated by the physician were used as new constraints for PlanIQ/AP. The beam energies chosen were primarily 6 MV, with two patients (2/20) using 10 MV, and computed using collapsed cone convolution (CCC) dose engine on a 2 mm dose matrix. Heterogeneity correction factors were enabled for all cases. CCC dose calculations were used in all cases. The treatment planning and comparisons were made retrospectively for patients that previously had re‐irradiation plans completed and treated using either an Elekta Versa or Synergy linear accelerators. Per departmental standards, all planning times were recorded via quality checklist (QCL) items to verify the overall length of the planning process.

**Fig. 1:**
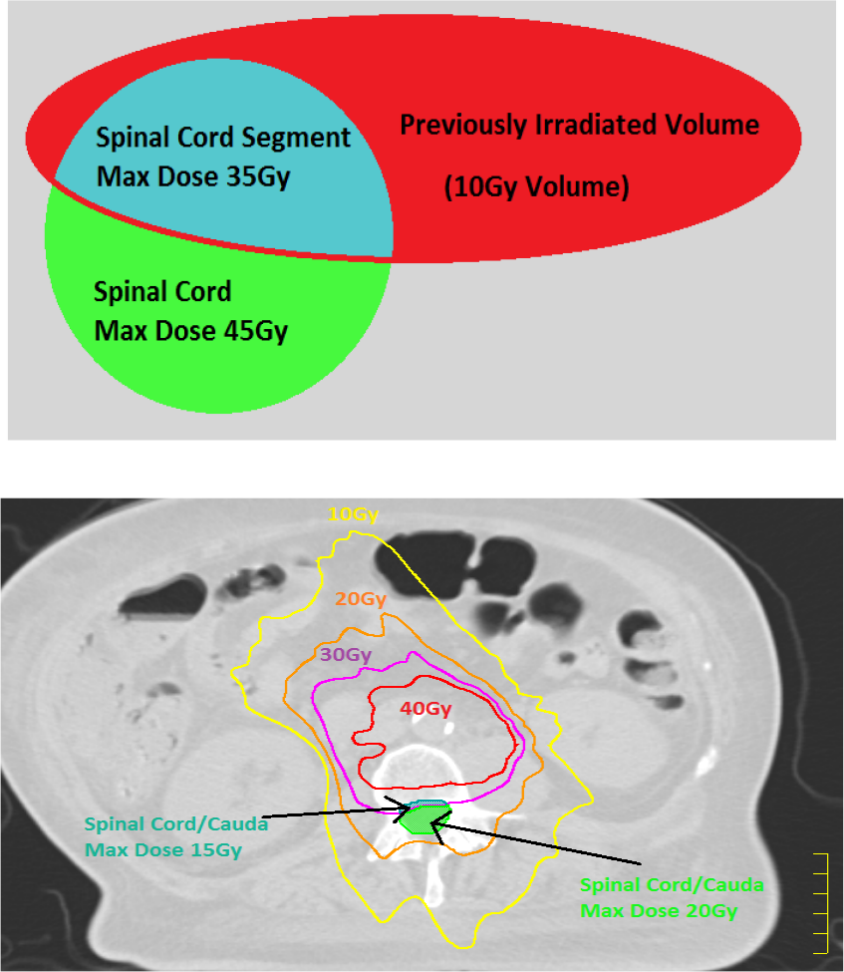
Representation of the means by which planners segment a critical structure based on the previously delivered doses.

The previously delivered doses were accounted for through an MIM dose accumulation which were then sent to Pinnacle as a contour set, providing a surrogate for the dose cloud which cannot be summed in PlanIQ at the time of this research. Proximal OAR overlapping the initial sites of radiation were segmented into contours that corresponded to the remaining dose they could receive without violating the chosen dose threshold. An oversimplified example being a region of spinal cord that previously received 10 Gy would be segmented out as “Spinal Cord MAX 35Gy,” thus reflecting the remaining dose that could be delivered before exceeding the generally accepted tolerance dose [Fig. 1].^19^ All overlapping OAR segments were entered into AP as a way of informing the planning system of the previously delivered dose and the strict constraints that would be necessary to accomplish the desired re‐irradiation. Treatment planning objectives were entered into the AP window without modification from the physician’s request. For proximal OAR without outlined objectives, QUANTEC and/or AAPM task group (TG‐) guidelines were used to set preliminary planning objectives for PlanIQ benchmarking. With all dose objectives in Pinnacle AP, optimization objectives were then exported directly to PlanIQ to begin the *Feasibility Analysis of Goals*.

**Fig. 2:**
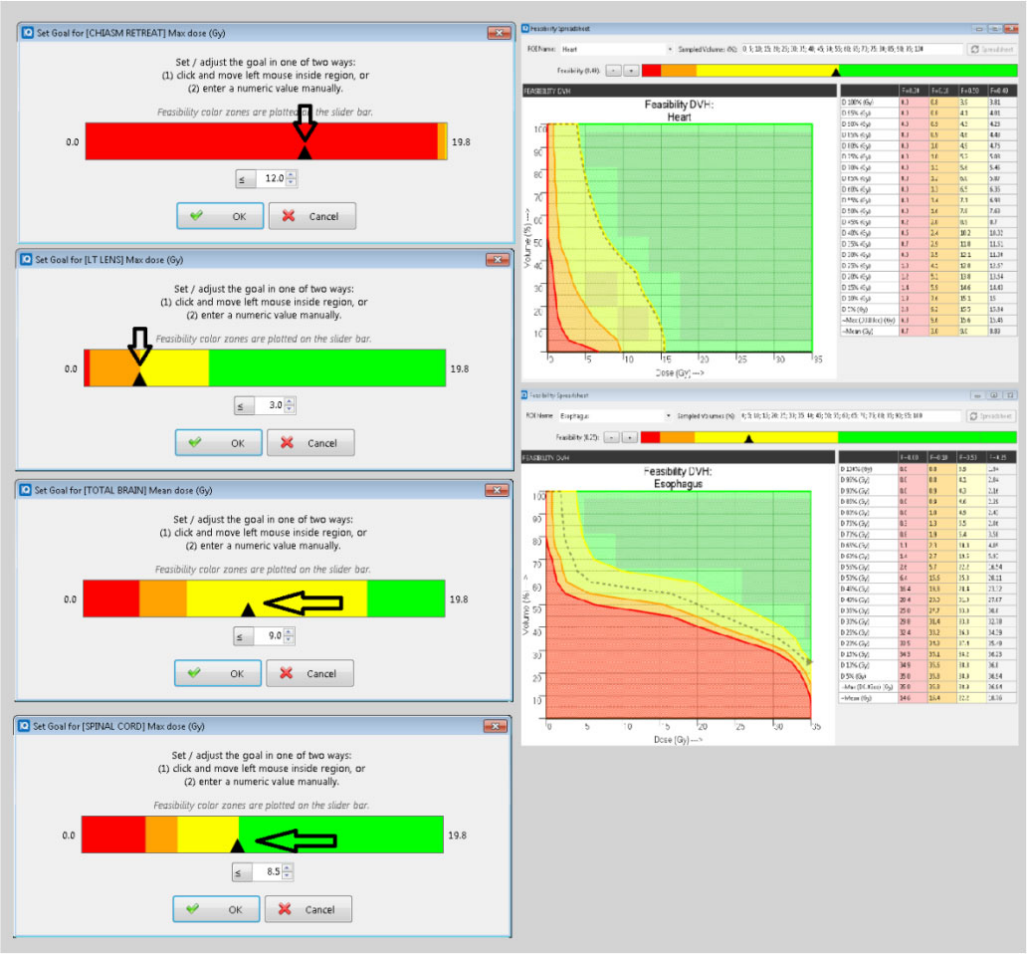
The left‐hand column represents the template described in *methods and materials*. Continuity in planning approach was necessary given the degree of variation between the retrospectively planned cases. The right‐hand column demonstrates feasibility DVHs (FDVHs) and the respective F‐Values for a given curve.

Planners are able to manipulate feasibility histograms within PlanIQ to refine specific dose objectives previously imported. Having been shown to increase plan quality, critical evaluation and manipulation of FDVHs give the planner crucial knowledge of how to approach a challenging scenario where dose constraints are of upmost importance.^20^ In cases of re‐irradiation, it is not uncommon to see critical structures, such as the spinal cord, overlapping both a previously irradiated volume and the newly delineated target. When this occurred, PlanIQ could return our requests as “Impossible” (Feasibility (F) < 0.0) or highly “Difficult” (F < 0.1). These requests needed to be met however, and therefore saw the “Compromise” function turned off within Pinnacle’s AP. Some objectives were determined to be “Challenging” (F = 0.11–0.5) while other objectives, seemingly easier to achieve requests, were deemed “Probable.” All “Probable” (F = 0.5–1.0) values were pushed by the user to barely inside the “Challenging” region (F = 0.49). Objective values thought to be “Challenging” were adjusted into the lower 50% of the corresponding range (F = 0.25), while “Impossible” and “Difficult” objectives were left unchanged [Fig. 2]. Once all adjustments had been made, there were no remaining objectives with the green “Probable” designation. Lastly, revised objectives were exported back to Pinnacle to begin the AP optimization process. This procedure became the template Seidman Cancer Center would pioneer, validate, and by which all patients were planned under this research.

## 4 Results & Discussion

A side‐by‐side comparison of isodose lines and dose–volume histograms (DVHs) was performed by the researching physicians to establish whether the PlanIQ‐AP generated plan was acceptable. Once reviewed and/or approved, all dose information was recorded for target volumes, critical OAR, dose overlap regions, and all contoured/segmented areas of interest.

Not only does PlanIQ provide physicians and dosimetrists with pre‐optimization insight into generating and pursuing realistic treatment objectives in these cases of complex re‐irradiation, but it can also provide feedback that has the potential to drive important decision‐making surrounding planning prescriptions. Historically, blind optimization would be performed via trial and error until it was determined one prescription would or would not be achievable. This study found that the feasibility software provided sufficient information to inform whether one prescription would be achievable at the onset of planning, saving significant planning time, and allowing dosimetrists to focus on achieving outlined goals while simultaneously delivering acceptable levels of prescription dose coverage to delineated target volumes.

Every re‐irradiation case poses unique challenges that theoretically may not be able to be pulled from a knowledge‐based or library‐based planning solution. Whether the challenges are due to the previously delivered doses or the modality in which it was delivered (i.e., 3DCRT, VMAT, CyberKnife SRS, GammaKnife SRS, TomoTherapy, or even brachytherapy), PlanIQ and the aforementioned planning template offer a comprehensive manner in which to account for planning scenarios with high variance. Concomitantly, the AP component of this integration further reduced variability by ensuring continuity in the optimization process.

Across 20 patient trials, the effectiveness of PlanIQ in predicting whether a complex re‐irradiation was possible was 100% (20/20) cases. Six (6/20) cases returned an “Impossible” feasibility for at least one of the planner’s requests. This indication was interpreted as the plan would not be achievable under 100% target coverage conditions and therefore necessitated the generation of a modified target for optimization, allowing under coverage within a given region. All treatment plans were evaluated by the researching physicians, and depending on institutional standards, 95% prescription isodose coverage was considered acceptable. For the patients who initially returned an “impossible” feasibility, all returned a second feasibility of “possible” once the optimization target volume was generated.

It should be noted that a small margin can be added to proximal OAR and, under re‐irradiation conditions specifically, subtracted from the PTV in order to attain a planning target structure. This allows AP to generate a steep dose gradient toward the structure whose dose objective must be observed over target coverage, such as a spinal cord approaching threshold dose. Furthermore, the six cases utilizing optimization target volumes for PlanIQ‐AP benchmarking and optimization had final plans determined to be of equal quality when compared to the manual plan. Similar to the findings of,^21^ optimization of OAR close to targets, or overlapping in the case of re‐irradiation, presented planners with an opportunity to benefit greatly from manipulating FDVHs for these segmented OAR.

**Fig. 3:**
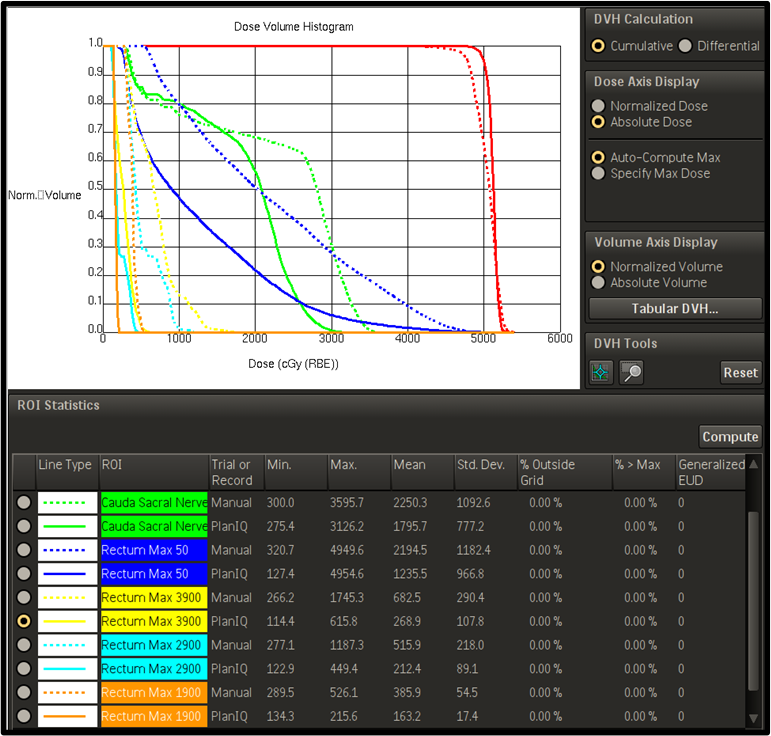
A Pinnacle‐rendered DVH example of how PlanIQ and manual planning compare when evaluating the direct dose overlap regions as segmented for a case of pelvic re‐irradiation.

**Fig. 4:**
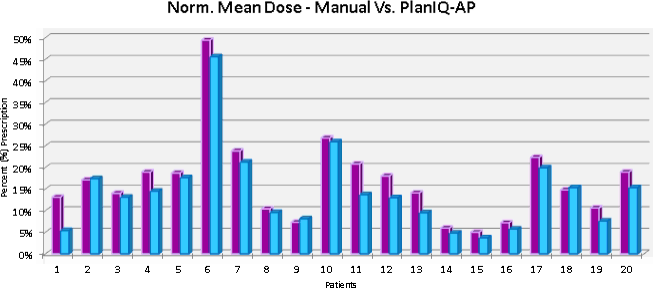
Manual in Purple (left‐sided bars) – PlanIQ/AP in Blue (right‐sided bars). Side‐by‐side graphical comparison of normalized mean dose to ***all*** proximal OAR.

OAR within regions of direct dose overlap saw improvements in 17 (17/20) patients, with the remaining three patients exhibiting similar, or unremarkable, improvements compared to the manual plan. Average maximum point doses to critical OAR for plans utilizing PlanIQ‐AP saw improvements ranging from as low as 60 cGy to as high as 5 Gy (brain) and an overall improvement of eight (8%) percent when compared to manual planning. These findings were found to be statistically significant (*P* < 0.06). Additional PlanIQ‐AP improvements, observed in patient example [Fig. 3], in max point dose and integral doses to proximal tissues and OAR can be found within specific dose overlap regions. Additional significant comparisons (*P* < 0.05) of normalized mean dose to proximal OAR were found to exhibit an overall 5% improvement through the use of PlanIQ‐AP [Fig. 4]. Utilizing PlanIQ’s feasibility feedback into ideal scenarios and AP’s iterative planning approach to optimization, the data collected herein suggests that it was in fact possible to drastically reduce both max point doses and integral doses to proximal OAR in a swift manner.

**Fig. 5:**
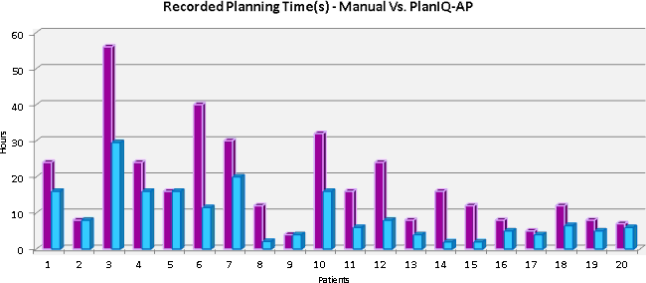
Side‐by‐side graphical comparison of recorded planning times.

It should also be noted that this process, across 17 (17/20) trials, was performed faster than manually iterating a treatment plan that met the outlined treatment goals [Fig. 5]. Three (3/20) trials required an equal or unremarkably extended amount of time for planning through PlanIQ as the manual plan had initially taken. While not the primary metric in our course of study utilizing PlanIQ and AP, it was clear from the recorded treatment planning timelines that once feasibility analyses and PlanIQ templates were implemented for these re‐irradiation cases, some cases did exhibit major improvements in dosimetry planning efficiency. These improvements to efficiency can be observed in Fig. 5. The largest planning time disparity was exhibited in a simultaneously integrated boost (SIB) re‐irradiation pelvic case where the PlanIQ‐AP was completed just over a day (28 hours) faster than the original plan. The time savings exemplified in Fig. 5 was found to be statistically significant (*P* < 0.05) in addition to the aforementioned dosimetric advantages. An average time savings of 8.7 h was calculated from the research population. These findings corroborate a previous study where it was found that both experienced and inexperienced planners gain significant time savings, 2.4–3.3%, respectively, through the use of dose prediction and feasibility models for planning.^22^

For patients who may eventually complete multiple courses of radiotherapy, reducing the maximum point doses and integral doses to proximal OAR allows the radiation oncologist the ability to re‐treat any given area, should the need arise. This proactive manner of thinking and treatment planning can not only preserve quality of life in the here and now, but it can mean the difference in whether or not a patient can successfully receive re‐irradiation of curative intent without possible dose de‐escalation. Furthermore, this study illustrates that the union of PlanIQ and AP does provide ideal sparing opportunities through informed treatment planning.

As discussed earlier, feedback gained through FDVHs assists planners in visualizing how the desired prescription, patient‐specific anatomy, critical structures, and target volumes interact within dose overlap regions. Up‐front segmentation of these regions is necessary, and the processes performed by PlanIQ are far quicker than manual trial and error planning. Even manual planning post‐PlanIQ would be more efficient than generating a manually optimized plan from start to finish, speaking to the scope of PlanIQ’s applicability.

Currently, PlanIQ does not support dose summation between a previously delivered plan, stored in DICOM format (MIM, Pinnacle, etc.), and the feasibility calculation. If PlanIQ were capable of generating a composite in this manner, the new model‐based calculation and the previous plan in DICOM format, it would eliminate the need to segment OAR into surrogate planning structures based on previous dose overlap, vastly improving the time it takes to segment OAR by hand. At this time, the research team believes this lack of functionality to be the most significant study limitation. Should this functionality be added in future versions, however, it would mitigate the potential for human error by further reducing the number of direct interactions planners would need to make in order to prepare PlanIQ objectives for export and analysis.

The second limitation of this study arose from a majority of the research team members pursuing new career opportunities at various healthcare organizations and/or academic settings shortly after the research had concluded. While additional investigation would provide greater generalization of results, specifically comparing PlanIQ‐AP re‐irradiation cases against those of outside organizations and/or partners, such opportunities are not available at this time. Consequently, this paper recommends that future studies consider the planning and examination of cases across multiple treatment centers and/or facilities to validate the template developed and presented by University Hospitals Seidman Cancer Center.

## 5 Conclusion

Emerging technologies continue to enhance practitioner’s abilities to provide patient‐specific treatment options, with the integration of PlanIQ and AP representing a milestone in the planning of complex re‐irradiation cases. Based on the initial findings of this study, it is clear that PlanIQ‐AP effectively provides the necessary feedback to facilitate physician decision‐making and eliminate blind optimizations through trial and error. Additionally, findings also suggest significant reductions in integral mean doses to proximal OAR as well as maximum point doses within critical dose overlap regions. To our knowledge, novel planning solutions that can adequately contend with such a wide array of re‐irradiation cases have not been fully realized and/or examined within the scientific, peer‐reviewed, setting. To that extent, it is the introduction of model‐based prediction tools into AP that has yielded positive outcomes in treatment plan generation and workflow efficiency.

## Conflict of Interest

While University Hospitals Seidman Cancer Center uses Elekta linear accelerators, MOSAIQ record and verify EMR, Pinnacle treatment planning system, and MIM, we have no conflict of interest to disclose.
